# Contact Trees: Network Visualization beyond Nodes and Edges

**DOI:** 10.1371/journal.pone.0146368

**Published:** 2016-01-19

**Authors:** Arnaud Sallaberry, Yang-chih Fu, Hwai-Chung Ho, Kwan-Liu Ma

**Affiliations:** 1 Montpellier Laboratory of Informatics, Robotics and Microelectronics, Paul Valéry University of Montpellier, Montpellier, France; 2 Institute of Sociology, Academia Sinica, Taipei, Taiwan; 3 Institute of Statistical Science, Academia Sinica, Taipei, Taiwan; 4 Department of Computer Science, University of California at Davis, Davis, California, United States of America; Melbourne School of Population Health, AUSTRALIA

## Abstract

Node-Link diagrams make it possible to take a quick glance at how nodes (or actors) in a network are connected by edges (or ties). A conventional network diagram of a “contact tree” maps out a root and branches that represent the structure of nodes and edges, often without further specifying leaves or fruits that would have grown from small branches. By furnishing such a network structure with leaves and fruits, we reveal details about “contacts” in our *ContactTrees* upon which ties and relationships are constructed. Our elegant design employs a bottom-up approach that resembles a recent attempt to understand subjective well-being by means of a series of emotions. Such a bottom-up approach to social-network studies decomposes each tie into a series of interactions or contacts, which can help deepen our understanding of the complexity embedded in a network structure. Unlike previous network visualizations, *ContactTrees* highlight how relationships form and change based upon interactions among actors, as well as how relationships and networks vary by contact attributes. Based on a botanical tree metaphor, the design is easy to construct and the resulting tree-like visualization can display many properties at both tie and contact levels, thus recapturing a key ingredient missing from conventional techniques of network visualization. We demonstrate *ContactTrees* using data sets consisting of up to three waves of 3-month *contact diaries* over the 2004-2012 period, and discuss how this design can be applied to other types of datasets.

## Introduction

Social networks, which are composed of actors (or nodes) and their connections (or edges), have been the subject of a very dynamic field of study for decades [1–3]. As major problems facing humanity in the twenty-first century are political, economic, and social in nature, many social phenomena result from interactions among people, institutions, and markets [3, 4]. Such interactions among various actors at different levels strongly call for interpreting social facts from a relational or structural perspective. Following the rapid rise of social media, particularly online social networking sites such as Facebook and LinkedIn, the structures of social networks have become even more complicated and difficult to understand. With the help of emergent visualization technologies, network researchers have more tools available for identifying the key properties of large sets of network data [5–8].

Most existing methods and tools consider social networks as a whole, relying on a global or complete network approach. As comprehensive as it can be, a global approach tends to leave out or downplay some essential aspects of social relationships. Few studies of complete networks, for example, focus on age and gender distributions of social ties, which can be more easily examined with a local approach. Although both global and local approaches can help explain the complexity and dynamics of social networks, egocentric network representations reveal patterns and trends that global representations fail to highlight [9, 10].

A local approach makes it possible to represent not only the connections among actors but also the characteristics of actors. To highlight the overall patterns of such connections, visualization tools often rely on tree-like network diagrams that use nodes (dots or circles) to represent actors, and edges (lines) to represent connections or linkages. These contact-tree diagrams, however, typically stop at the connection level and lack further details about the elements upon which a connection is built: contacts or social interactions. As social relations are created and maintained by *interactions* among actors, a visualization tool that fails to capture such interactions cannot fully display the dynamics of social networks. Existing visualization tools thus do not meet the needs of researchers who aim to explain how social interactions or contacts impact the formation and evolution of egocentric networks. In this paper we show how we resolve this issue with a new visualization design.

We present *ContactTrees*, a new egocentric visualization design that can help researchers assess social interactions, compare interpersonal relationships, and make hypotheses about patterns or trends of contacts in everyday life. The design is based on a botanical tree metaphor. The main idea is to use the features of a tree (the structure of its branches, leaves, fruits, colors, *etc.*) to map the properties of social interactions. This design fits well for visualizing many properties of egocentric networks. We present an application, using specific datasets, to highlight some trends and hypotheses. The datasets contain up to three waves of 3-month *contact diaries* that were collected between 2004 and 2012 [11] (*i.e.*, lists of one-on-one contacts for three periods of 3 months each).

Our first contribution to social network visualization lies in the local perspective. While most social network visualizations adopt a global approach, we take an egocentric approach to highlight selected aspects of social interactions. The previous works devoted to local approaches mainly aim to convey the distance between the person of focus and his or her network neighbors [12–14], without any means to characterize these neighbors. Our approach aims to help identify individuals’ overall ties and contacts with their network neighbors at a glance. Comparing the properties in several *ContactTrees* further reveals trends about these individuals’ social lives. By looking at an individual’s *ContactTrees* from different time periods, one can also speculate about the individual’s personal and social life stories.

The second contribution comes from displaying leaves and fruits that symbolize interactions, contacts, or meetings among persons. While nearly all network graphs include nodes (actors) and edges (links or ties), our visualization moves a step further by incorporating critical information about each specific contact or meeting into the network. Extending the use of “contacts” as the building block of a network structure [15], we perceive social networks not only in terms of how actors are connected, but also in how such connections vary contact by contact. Because a relation is essentially established by a series of contacts between two actors, showing the properties of such fundamental building blocks by leaves and fruits greatly helps us understand relations and networks. In other words, our design employs a bottom-up approach that resembles a recent attempt to understand subjective well-being by means of a series of emotions [16]. Such a bottom-up approach to social-network studies decomposes each tie into a series of contacts, which can help deepen our understanding of the complexity embedded in a network structure.

The nature of our design makes it possible to map various properties at both tie and contact levels. The specific mapping can be selected according to the intended tasks. Another strength of our design lies in the botanical metaphor used in such a context. In addition to producing attractive representations, our design is extensible, because it is relatively easy to add new glyphs showing other aspects of the dataset. With these strengths, *ContactTrees* should appeal to social-science researchers who wish to take advantage of visualization tools that readily help them pinpoint critical features embedded within the multilevel data in social networks.

## Related Work

In this section, we review the current state of relational data visualization, realistic botanical tree creation, and data visualizations based on botanical metaphors.

### Visualizing Relations

Most approaches to visualizing relationships are based on graphs, where nodes represent persons and edges represent the relations among them [17]. Such approaches are closely related to the domain of graph drawing, which focuses on algorithms that help embed graphs in readable ways (see [18, 19] for an introduction). Although the sizes of most social networks generate highly cluttered drawings, researchers of information visualization have developed many techniques to simplify representations (see [20, 21] for an overview). Some of the most powerful techniques involve clustering and navigation, such as *TopoLayout* [22], *ASK-GraphView* [23–26], and edge bundling [27], or hybrid drawing methods like *NodeTrix*, where some communities of the network are displayed as matrices [28].

Navigating through networks from local views has also been addressed. For example, methods presented in [12] and [29] are based on tree layouts that allow users to explore a network from a given node. Van Ham and Perer also proposed a large graph visualization technique [30] based on the computation of degrees of interest, in order to guide the user during the navigation.

More specific techniques have also been proposed. Jeffrey Heer and Danah Boyd designed and implemented a graph visualization tool for online social networks [13]. Baur *et al.* proposed a software that includes graph visualizations and many network analysis metrics and techniques [5]. Fisher and Dourish [31] developed applications based on collaboration network visualization to help coordinate and manage these collaborations. *ContactMap* [32] is an interface for visualizing groups of an individual’s personal contacts. A similar approach has been proposed in [33]. With this tool, users can navigate through overlapping groups of friends. The visualization of more structured relationships, such as genealogies, also has been addressed [34–36].

Another network-visualization approach adopts a tree-like structure. This approach is used when networks are composed of strictly hierarchical relationships, in which each element is subordinated to one and only one other element. The trees in this approach, however, are not to be confused with our *ContactTrees*. Although both are derived from the botanical metaphor, the underlying structures are quite different. Before providing a quick overview of tree visualizations, it is important to comment on the tree structure itself. Consider a graph in which vertices are the persons of a social network and the edges are their links. Constructing a spanning tree from a given person produces an egocentric structure that reveals the distance between this person and other persons in the network.

This technique is inspiring but not sufficient for our purpose. First, not only do we focus on how network members are directly connected to a focal person, we also aim to map various properties of these members. Second, in addition to such relationships and properties, we can further distinguish the attributes of social interactions between each network member and the focal person, contact by contact. There are three main approaches for the visualization of trees. According to the paradigm of node-edge diagrams, persons are represented by small shapes and relations by lines. A good introduction to the techniques is given by two books on graph drawing [18, 19] and the *treevis* website (http://treevis.net/) [37]. Persons can also be represented by areas and relations by the positioning of these areas. This is the case of *Icicle Plots* [38], *Information Slices* [39], and *Sunburst* [40]. The third approach visualizes tree elements as nested areas. Two methods have been proposed to create such maps: (1) dividing the plan recursively (a detailed overview by Ben Shneiderman, and updated by Catherine Plaisant, can be found at [41]); and (2) positioning leaves along space-filing curves [42].

### Visualizations based on Botanical Metaphors

Following the seminal papers of Ulam [43] and Hondal [44], computer modeling of trees has been an active area of research. The Previous Work section of [45] provides a good overview of results in this area. These methods attempt to model the way real-world botanical trees grow. Therefore, they do not reflect a structure defined a priori. Moreover, most of them incorporate random settings, which make them incompatible with our purpose: Our visualization must incorporate a pre-defined structure and be based on a deterministic algorithm to facilitate comparisons.

One of the few preexisting visualizations based on botanical metaphors is the representation of trees (*i.e.*, hierarchical data structure) as botanical trees [46]. These trees are 3D, while ours are 2D. A recurrent problem of visualizations in 3D lies in occlusion, while a 2D approach is more suitable for comparison. Our visualizations are also much simpler because of the input structure of the data, and thus they are easier for average users to use and interpret. Chlan and Rheingans also proposed a method to visualize hierarchical structures as botanical trees [47]. They combine this visualization with a branch cross-section visualization that represents properties shared by groups of elements. *Notabilia* is a visualization of *Wikipedia* discussions with a nearly identical design with a botanical metaphor [48], which shows similarities of the sequences of deletions in *Wikipedia* discussions. Xiong and Donath proposed a flower metaphor to visualize an individual’s behavior in online interactions [49]. They show how their design is helpful in finding information and comparing individuals. Their design is simpler than ours, and they cannot map as many attributes as we want to for our purposes.

## Overview: Visualizing Contacts

Like other tools of tree visualization, our *ContactTrees* help visualize social relations in egocentric networks. More importantly, they further capture the properties of each unique interaction, or contact, within a relation. When used properly, *ContactTrees* present overall, succinct tie and contact properties of complex social structures embedded in “egocentric contact networks”, an alternative network reconstruction emerging in the latest comparative studies [50].

**Fig 1:**
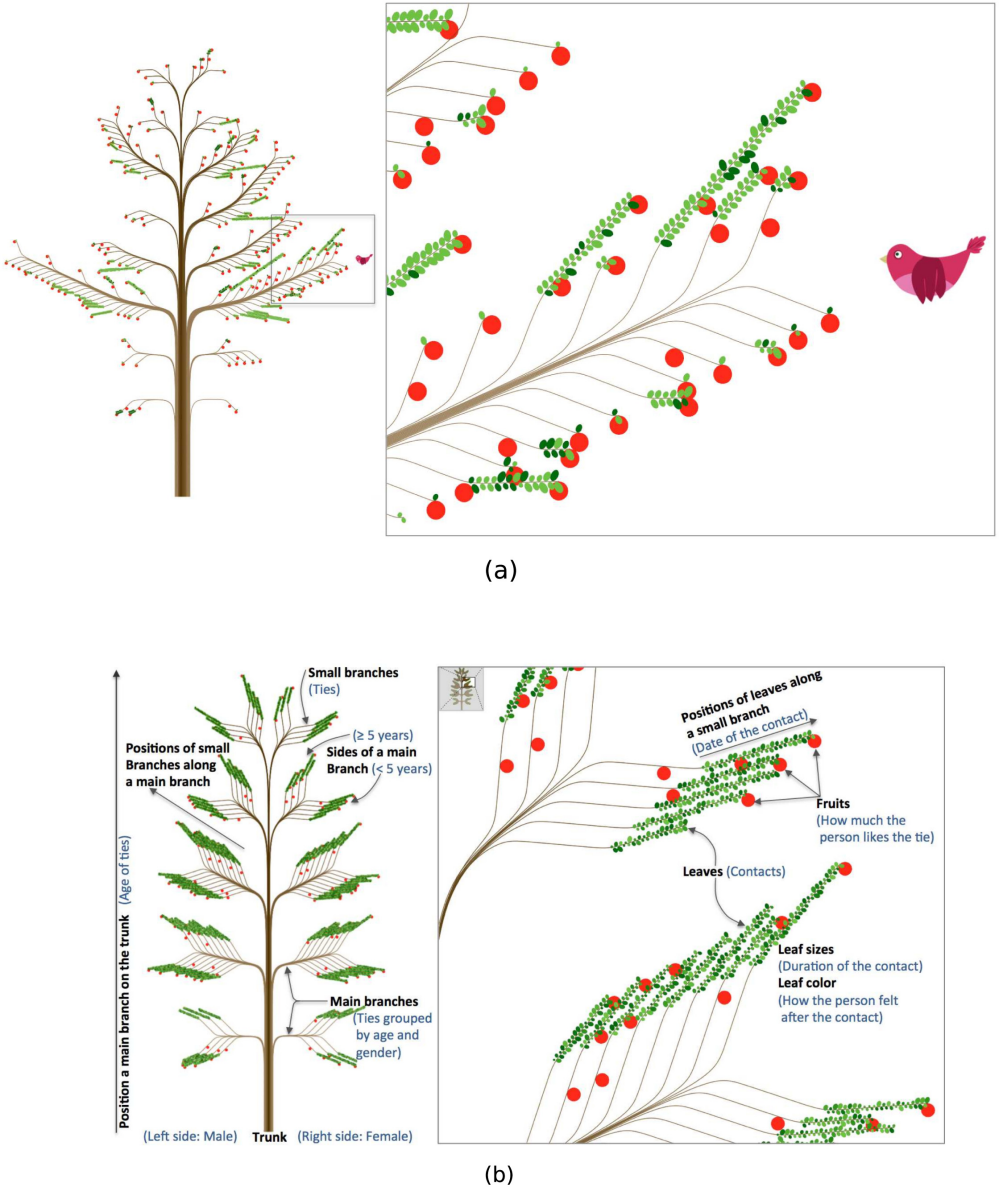
Mapping properties on tree features. (a) A *ContactTree* that represents the ties and contacts of a 25-year-old woman. Each small branch is a tie, with male ties on the left side of the trunk and female ties on the right. Leaves represent contacts with these ties. (b) Features of the tree on which we map properties (black labels) and an example of mapping (blue labels).

To highlight such visualization effects, Fig 1a shows a *ContactTree* representing the ties and contacts of a 25-year-old woman (*i.e.* the persons this woman met during a given period and the contacts between the woman and these persons). With a conventional tool of network visualization, the tree metaphor would have stopped at the branch level, which indicates interpersonal ties. Our design, in contrast, furnishes each of these branches with various kinds of leaves, thus effectively reflecting contacts within each of the interpersonal ties.

Centered around the primary goal of visualizing contacts, we develop a design based on the botanical metaphor following four principles. First, the design should allow users to map as many tree features as possible. Second, it should make it easier to remember the main aspects of a tree than the main aspects of a more conventional visualization. Third, the tool should enable one to compare the properties of ties and contacts, and to identify how such ties and contacts evolve over time. Fourth, the design should be effective and efficient in facilitating the adoption of information visualization techniques in social sciences.

A 2D botanical tree structure can unambiguously hold many properties. Main branches growing from the trunk, for example, are on either the left or the right side of the trunk. They also have a y-position on the trunk, an angle, a length, *etc.* All of these features can be used to symbolize properties. We have selected some of them, as described in the next section. We then present an example of mapping.

### Features of the Botanical Structure Used to Map Properties

First of all, a tree can be seen as a structure composed on a trunk, which is divided into several part to form main branches, which are further divided into smaller branches, *etc.* Starting from this observation, an intuitive approach consists of representing ties as the smallest branches (see black labels on Fig 1b). Using this approach, a small branch holds exclusive properties of a tie, while bigger branches represent common properties of the ties shared by the small branches growing out of them. Finally, the trunk represents the whole set of relationships.

The starting point of each main branch growing up from the trunk can be characterized by two parameters: its position along the trunk and the side of the trunk on which it is located (see Fig 1b). The position along the trunk is ranked on an ordinal scale, and the side of the trunk is a boolean one (either right or left). These two properties characterize the set of ties represented by the main branches.

The same approach is also valid for the main branches. The position along the main branch and the side (above/below) from where the smallest branch is starting can be used to represent properties (one ordinal and one boolean). The boolean property depicted by the side of the main branches, however, is less salient than the one represented by the side of the trunk. Users tend to pay less attention to it, and thus it should be reserved for less important properties. Because the smallest branches start from the main branches, we could also divide the main branches into smaller branches holding several ties, and so on, to display more properties, but we prefer to address this task as a future work. Indeed, deeper and more complex decompositions in visualization may or may not help reveal ties and contacts more clearly, or allow end users to apply their own data to the design as easily.

In our design, each small branch represents a tie, while leaves and fruits symbolize attributes of contacts. Each leaf/fruit also holds four features on which we can map a property: color, size, position along the branch, and the side of the branch from which it is growing (see Fig 1b).

Further considering the properties one can map on a *ContactTree*, we see first that there are two boolean ones (side of the trunk, side of the main branches) and two ordinal ones (position along the trunk, position along the main branches) for grouping the ties. Then for each tie, we have two numeric properties (leaves and fruits). If they encode specific elements, two numeric properties of these elements (color, size) can be shown, as well as an ordinal property (position along the small branch) and a boolean property (side of the small branch).

### Example of Mapping

Many properties of ties and contacts can be mapped using the tree features described above. The example of mapping presented here is based on *contact diaries*, a major approach of data collection in the social network literature [9, 11, 51]. A diary normally contains inclusive information about persons with whom the diary keeper has contact during a given period of time, their relationships with the diary keeper, and the situations of each one-on-one contact. Some diaries are more specific about certain properties. For example, contact situations might include how much the diary keeper likes a contacted person, or how the diary keeper feels after each unique contact. In this paper we use an actual dataset of *contact diaries*.

Blue labels in Fig 1b show an example of mapping. Here, branches lying on the left (right) side of the trunk are male (female) ties. Positions of the main branches along the trunk represent age groups (or age cohorts) of the contacted persons: ages 0 to 9 for the lowest main branch, ages 10 to 19 for the second-lowest branch, and so on. The tree drawn according to these principles so far resembles a population pyramid in terms of a gender/age structure diagram, thus linking our design to conventional demography as well. Furthermore, sides of the main branches indicate how long the diary keeper has known the contacted person (at least 5 years if a small branch is above the main branch, less than 5 years if it is below).

Each leaf is a contact between the focal person and the tie represented by the small branch from which it is growing. Leaves are ordered along a small branch by the date when the contact occurred (we do not distinguish the side of the small branch from which a leaf grows). The darker a leaf is, the better the person felt about the contact. The size of each leaf represents the duration of the contact. Finally, fruits signify how much the person likes the tie (no fruit means “not at all” or “not much,” 1 fruit means “somewhat,” 2 fruits mean “very much”).

Typical questions supported by our design are “Which gender is most present in a person’s relationships?,” “What is the age distribution of these relationships?,” “Who are the persons one prefers?,” “How do all these properties evolve for a given person?,” or “Are there some trends among the relationships of persons who have a child?” The reconfigurable interface described in the next section allows a user to select the attributes mapped into the *ContactTrees* according to the objectives of her analysis or presentation. Features supported by our approach can be divided into three main categories, each of which is further divided into smaller types of features:

1. **Global aspect of a** ***ContactTree***: Balance (side of the trunk, side of the main branches), Distribution of the values (positions along the trunk and along the main branches), Outliers in terms of the quantity of contacts (size of the small branches).
2. **Details of the ties of a** ***ContactTree***: Number of contacts (number of leaves), Qualities of the contacts (length and color of the leaves), Quality of a tie (number of fruits).
3. **Comparison of several** ***ContactTrees***: Trends among interpersonal relationships (tree shapes), Evolution of individual relationships comparing two time-steps (global tree shapes refined by local aspects of the trees).

In the Case Studies section, we will examine how it can be useful to compare persons and discover the trends among them.

### An Interface for Visualizing ContactTrees

A potential issue with our visualization lies in the burden of remembering visual encodings. This problem can be alleviated somewhat by implementing an interface that includes a legend. Using such a legend, social scientists who have tried our system have been able to interpret the *ContactTrees* quickly.

Which mapping to choose highly depends on the user’s purpose or the task to be performed. We provide a reconfigurable interface where the user can select mappings and explore a dataset on his own, finding the best perspective for conducting his tasks or comparing mappings if needed.

Our prototype has been developed as a plugin of the framework *Tulip* (http://tulip.labri.fr/TulipDrupal/) [52]. This framework includes a powerful data model and rendering system as well as many interaction techniques useful for our purposes. In particular, the user can zoom in/out using the mouse wheel or selecting a rectangle with the mouse, making it easy to access the details of small features like fruits or leaves. When zooming in, a small rectangle at the top left corner of the view shows the overall map, highlighting the area that one is viewing. In other words, the main area is devoted to the focus, while the rectangle shows the context. Another related technique available is a fisheye lens. The user can also move the map with a drag’n drop, search branches to select ties sharing a given property, and take snapshots.

## Algorithm

Our tree construction algorithm is based on three main steps as follows, as well as optional steps to show additional features.

### Step 1: Ordering Ties

**Fig 2:**
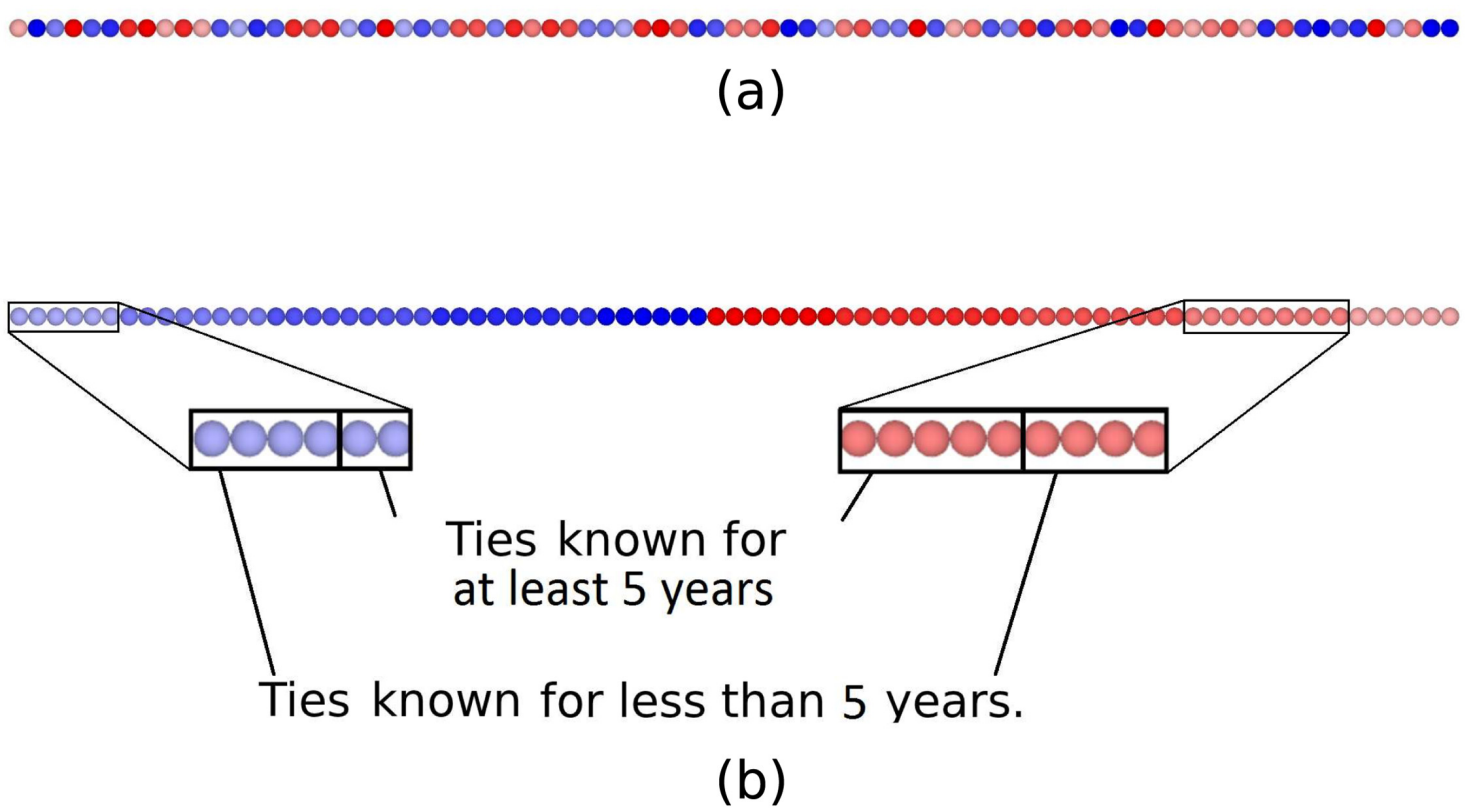
Ordering of the relations according to the mapping presented in Fig 1b. (a) Non ordered nodes. (b) Ordered nodes.

Our first step is to order the ties according to the three properties we want to take into account (see previous section). Fig 2a shows non-ordered nodes representing the ties. Blue nodes are the ties represented by branches on the left side of the tree (*e.g*., males in the example of the previous section); red nodes are the ties represented by branches on the right side (*e.g.*, females). Saturation depicts the properties corresponding to their positions along the trunk (*e.g.*, age groups). These colors are used to help clarify our method.

Nodes are then ordered as follows: Male nodes are first positioned on the left from the less saturated to the more saturated; female nodes are then positioned on the right from the more saturated to the less saturated. Nodes representing ties lying on the same main branch (same color and saturation, *e.g.*, same gender and same age group) are ordered according to the side of the main branch from which their small branches originate (*e.g.*, the number of years the person has known these ties), in increasing order for the left nodes and decreasing order for the right nodes. Fig 2b shows the result.

### Step 2: Drawing Branches

Our trees are made up of lines, with each line representing a tie. A line is at first a part of the trunk; then it becomes part of a main branch. Finally, the last segment is the small branch on which the leaves lie. In other words, the branches actually stem from the base of the tree, and the trunk is just the aggregation of branches.

**Fig 3:**
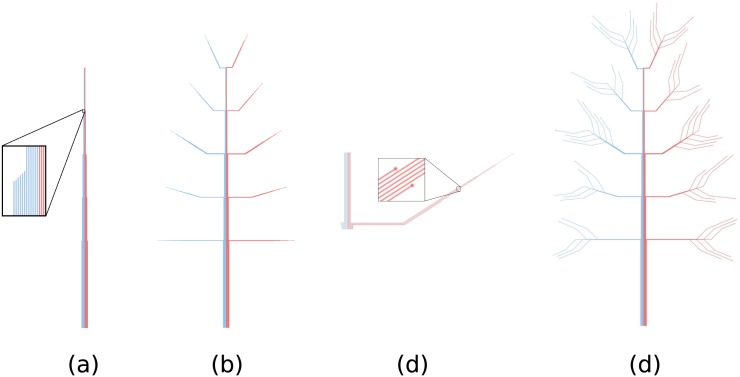
Trees are made up of lines. (a) Trunk: first segment of the lines. (b) Main branches: second and third segments of the lines. (c) Lines end uniformly on the upper/lower side of the branch according to the number of years the person has known the ties. (d) Small branches: fourth and fifth segments of the lines.

All lines start from the ordered nodes created in Step 1. Nodes form the basis of the trunk. The length of each line depends on the property mapped on the position of main branches along the trunk. In the previous example, an older tie is represented by a longer line (see Fig 3a, where gradient colors have been temporarily removed to ensure the visibility of small branches that were already light).

Then, the second and third segments are added (see Fig 3b). As these two new segments are positioned higher along the trunk (*i.e.*, when the contacted persons are older), the second segment gets shorter and the angle between the two segments becomes sharper. These two design choices yield a triangular structure that looks more like a botanical tree. The length of the third segment varies on the order computed during the previous step. The ties known by the person for at least 5 years are placed uniformly on the upper side of the branch, while those known less than 5 years are placed uniformly on the lower side (see Fig 3c).

Finally, two more segments (the fourth and fifth) are added to separate the lines from one another (see Fig 3d). The length of the fourth segment is a constant, as is the angle between segments. These constants do not display information but are required to obtain a botanical tree-like drawing. The length of the fifth segment depends on the number of leaves growing from the small branch. Thus, the more contacts a tie involves, the longer the fifth segment will be.

### Step 3: Adding Leaves and Fruits

Leaves are then added according to the side of the small branch upon which they have to lie. This side can represent a given property, as mentioned in the overview section. Fruits are all displayed on the lower side of the small branch in order to fulfill the botanical metaphor. The size and color of the leaves and fruits all depend on selected properties. As an example, the darkness of leaves in Fig 1b stands for how positively the person feels about a contact, and their size indicates the contact’s duration.

Lines are finally replaced by Bézier curves going through points uniformly distributed along them. Botanical tree-like colors are used. A gradient of brown on the trunk and branches shows the age range of the ties, highlighting the asymmetry between the right and left sides of the trunk. These design choices make our visualization look more like a botanical tree (see Fig 1a).

### Step 4 (optional): Adding More Features

A beneficial aspect of our visualization is its capacity for evolution. One can easily define new features that fulfill the botanical metaphor to highlight some additional aspects of a dataset. An example of this property is shown in Fig 1a. The *ContactTree* represents a person’s ties and the contacts this person has made with these ties. Mapping is the same as that described in the Overview section. As we will see in the Case Studies section, one purpose of our visualization is to allow more intuitive and direct comparisons between contacts and ties. In this case, displaying some properties of the person, such as gender, age and marital status, should help reveal trends among relationships. Of course, such properties instead could have been displayed as text near the *ContactTree*, but we dismissed this option for two reasons. First, it would not fulfill the botanical metaphor and would give the visualization an aesthetically unpleasant aspect. Second, a more intuitive feature could be used to clearly identify the focal person’s gender and age group. These two properties are already mapped for the ties by the side of the trunk and the positions of main branches. The same principles also should be used to map the focal person’s properties.

For that reason, we have introduced a bird (Fig 1a). The bird’s y-position indicates the person’s age: The bird is in front of the third main branch, so the person is between 20 and 29 years old, as are the other ties represented by this main branch. In the same way, the bird is on the right side of the trunk. This means that the person is a woman, as are all the ties on the right part of the trunk. Finally, we use two birds when the person is married and only one (as in Fig 1a) when he/she is not. Displaying married birds near the spouse of the person represented by the tree has also been considered. This design adds information and would be intuitive for married people, but would be misleading in the case of single persons.

The possibility of adding more features depending on the dataset is a particularly promising aspect of our visualization, and it is limited only by the designer’s creativity. While including additional features would make the visualization more attractive, it is also a good way to capture more properties. As an example, different birds could be used to highlight a handful of very special people.

## Case Studies

The main purpose of our visualization is to help network researchers and other end users compare both ties and contacts of several entities. Thanks to the Egocentric perspective, this comparison can involve properties of different persons, ties, and contacts. It also allows direct comparisons of how personal networks change over time. In this section, we use data from *contact diaries* to demonstrate how our design helps reveal such network structures and changes.

This study draws data from 11 data sets of 3-month *contact diaries* from 6 participants in Taiwan. Three sets of the diaries recorded all one-on-one interpersonal ties and contacts with three participants from March to May, 2004. Two more sets contained two waves of 3-month diaries, recorded by another participant in 2004 and 2008. The remaining six other sets covered three waves of 3-month diaries that two other participants kept in 2004, 2008, and 2012.

In all these diaries, the same key properties have been recorded or extracted from each of the contacts. These properties cover the situations of each contact (*e.g.*, duration and how the diary keeper felt about the contact), characteristics of each contacted person (*e.g.*, gender and age), and the relationships between each diary keeper and each contacted person (*e.g.*, how many years they had known each other, and how much the diary keeper liked the contacted person). Using the mapping techniques from our design makes it possible to easily identify patterns and trends of ties and contacts in the rich and complex information yielded from the *contact diaries*.

### Ethics Statement

All participants gave verbal informed consent. Like most other social science studies relying on surveys and in-depth interviews in Taiwan at the time of our data collection, our study followed the norm of collecting empirical data by obtaining the participants’ verbal consent, instead of written consent. We requested no written consent also because the study did not involve any medical, pharmaceutical, or physical procedure, and none of the participants belonged to any vulnerable groups. The verbal informed consent was recorded in the working logs kept by the project manager.

The consent procedure was neither reviewed nor approved by an ethics committee due to structural constraints. When we collected the contact diaries, no IRBs or ethics committees had been established in Taiwan to review and approve behavioral and social science studies for research ethics. In the early 2010s, the National Science Council started to help institutionalize such committees at universities and research institutes. As one of the earliest institutes that complied with the new policy, the Academia Sinica, where data collection of this study was based, started to review for research ethics in 2013 by its newly established IRB on Humanities & Social Science Research, or IRB-HS. Even though data collection of the current study did not have a channel for IRB approval at the time, as a pilot study of contact diaries it developed and led to standard procedures for human subject protection in two subsequent, larger diary studies (the corresponding author of the current paper, YCF, served as the PI in both research projects). Following the same procedures, the first subsequent large-scale diary study (a diary-based survey that collected contact diaries from a national representative sample of those ages 0-97) was later approved by the IRB-HS, Academia Sinica (in November 2013, AS-IRB-HS 13020). The second subsequent research project of contact diaries, which collected diaries on social media for up to 6 months, also adopted very similar procedures regarding research ethics and obtained approval by the same IRB-HS (in May 2015, AS-IRB-HS 13022).

Compared with these two IRB-approved subsequent studies, we collected a significantly smaller number of diaries. The ethics procedures covered both data collection and data processing in order to minimize any possible leaks of their personal information. First, we contacted the participants directly and trained them how to record the contacts by themselves. To further protect the privacy of the participants and those whom they had contacted, we instructed them to write down nicknames or other unique names that were not easily identifiable to others. Finally, all demographic and socioeconomic characteristics, as well as the features of the relationships and contacts, were coded into numbers by the participants themselves, who were assigned random identification numbers that appeared on the diary logs. Only the PI and the project manager checked the linkage for data cleaning, after which any personal information was delinked. As a result, all diary keepers and the individuals they had contacted became anonymous and not identifiable.

### The Mapping

Following the mapping procedures described in the Overview section, we present and compare several contact trees based on contact diaries taken by different individuals in different years. More than just displaying data, our visualization can be used to make hypotheses about a person’s social life and identify some trends among all the contacted persons in an egocentric network.

**Fig 4:**
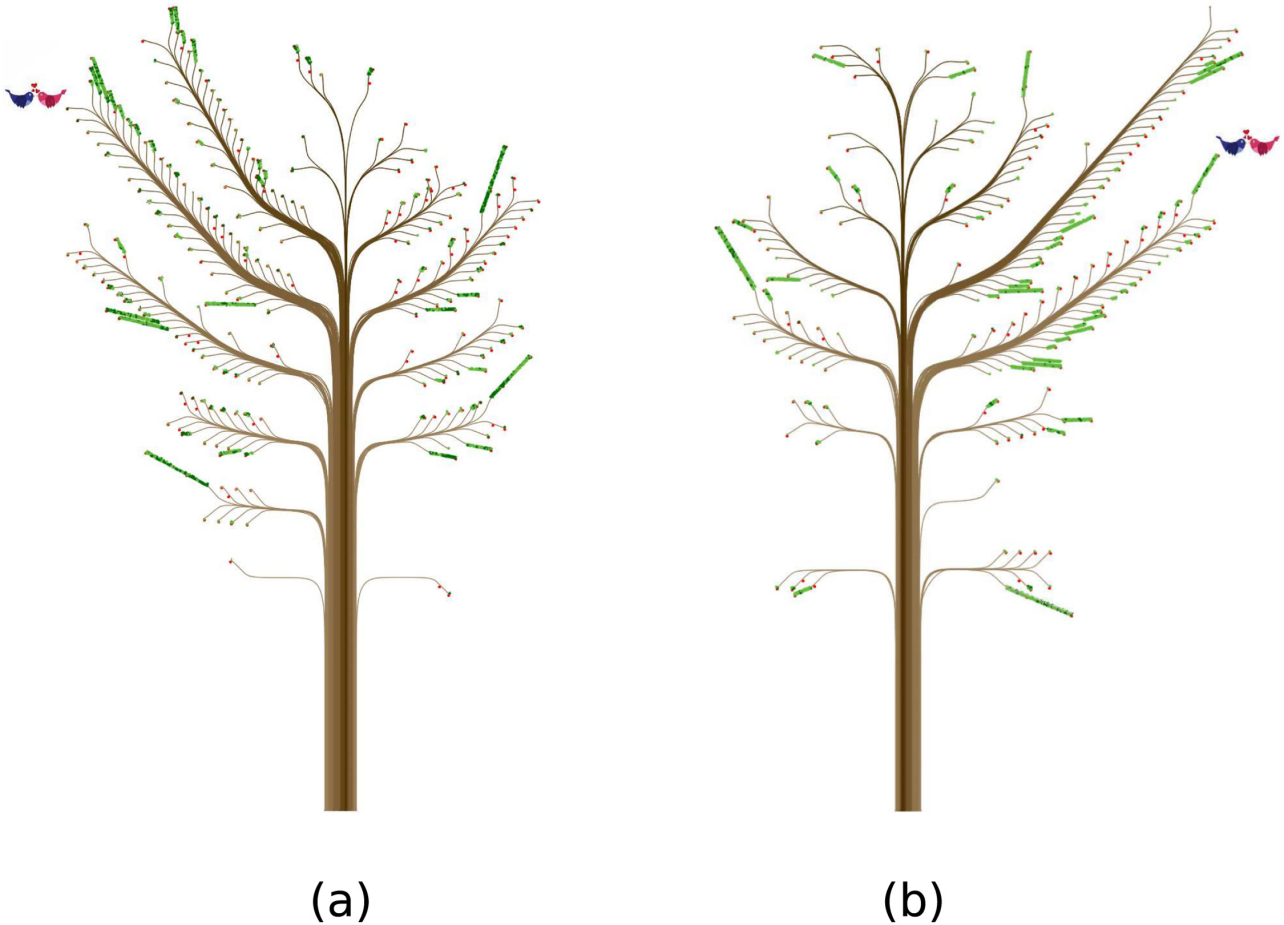
Two persons having many ties of their own gender. (a) represents a married man (S2 File) and (b) a married woman (S3 File). They are not a married couple.

Fig 4 shows two trees, each having a widespread structure. Each tree represents a married person (with a couple of birds, not a married couple in this case) having many ties of their own gender (on the same side as the birds), and one often-met tie from the opposite sex in the same age group in 2004. Each of these latter unique ties bears two fruits, which is the highest value for the property “how much the person liked the tie.” (See S1 File for details.) So, this particular tie is very likely the spouse. Sometimes, these kinds of trees also show often-met ties in the main branch of ties who are about 30 years younger than the person (*i.e.*, the second left main branch starting from the bottom of the left tree in Fig 4, and the first right main branch of the right tree). These ties also have two fruits. We can reasonably assume that they are the person’s children.

**Fig 5:**
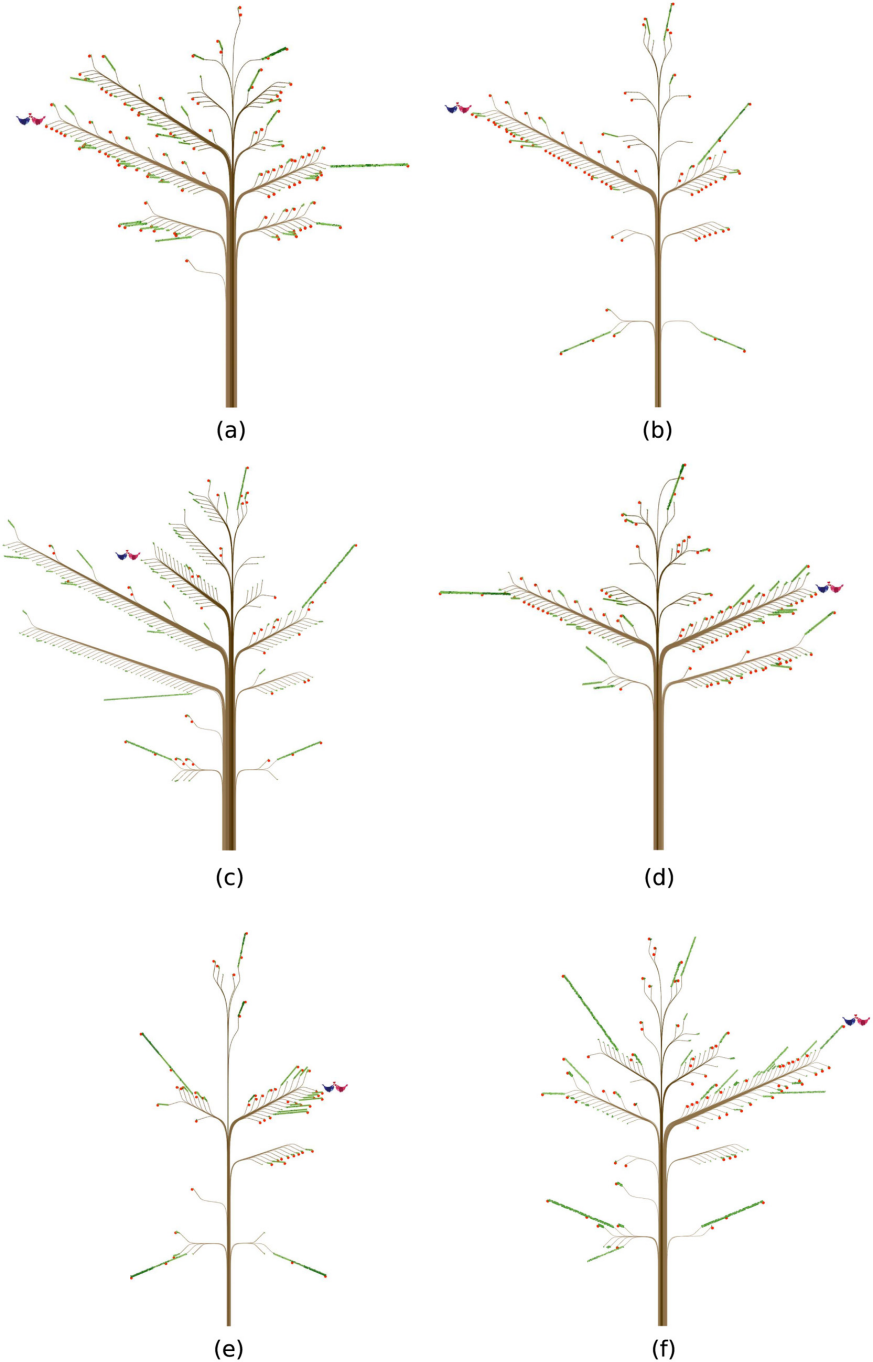
Does being a parent ruin social relations?. (a) and (d) represent two married persons (a man and a woman) with no children in 2004. (b) and (e) show the same persons four years later. They have two children in 2008. The numbers of their ties and contacts have decreased. (c) and (f) show the same persons in 2012. The numbers of their ties and contacts have increased, and the trees look pretty much the same as the ones back in 2004.

Fig 5 shows how a married couple’s ties and contacts changed in eight years. The husband’s starting properties were similar to those of the example in Fig 4. In 2004 the couple did not have children, and the husband’s ties and contacts were rich, particularly with those of the same gender (Fig 5a, S4 File). In 2008, both his ties and contacts shrunk significantly (Fig 5b, S5 File). Two new ties with 0-9 year olds emerged, however. These new ties were probably the couple’s children. In 2012 (Fig 5c, S6 File), the husband’s ties and contacts with several age groups of both genders increased, and the tree looks pretty much the same as the one back in 2004. The wife appears to have experienced very similar changes to those of her husband during this period: She first had abundant ties and contacts in 2004, which decreased markedly in 2008 with two new strong ties with 0-9 year olds, and then returned to a social life filled with many ties and contacts in 2012 (Fig 5d–5f, S7–S9 Files). This trend suggests a correlation between the size of the overall ties and contacts and the presence of young children: Having young children seems to reduce parents’ ties and contacts with others at first, but these ties and contacts may resume as the children grow up.

**Fig 6:**
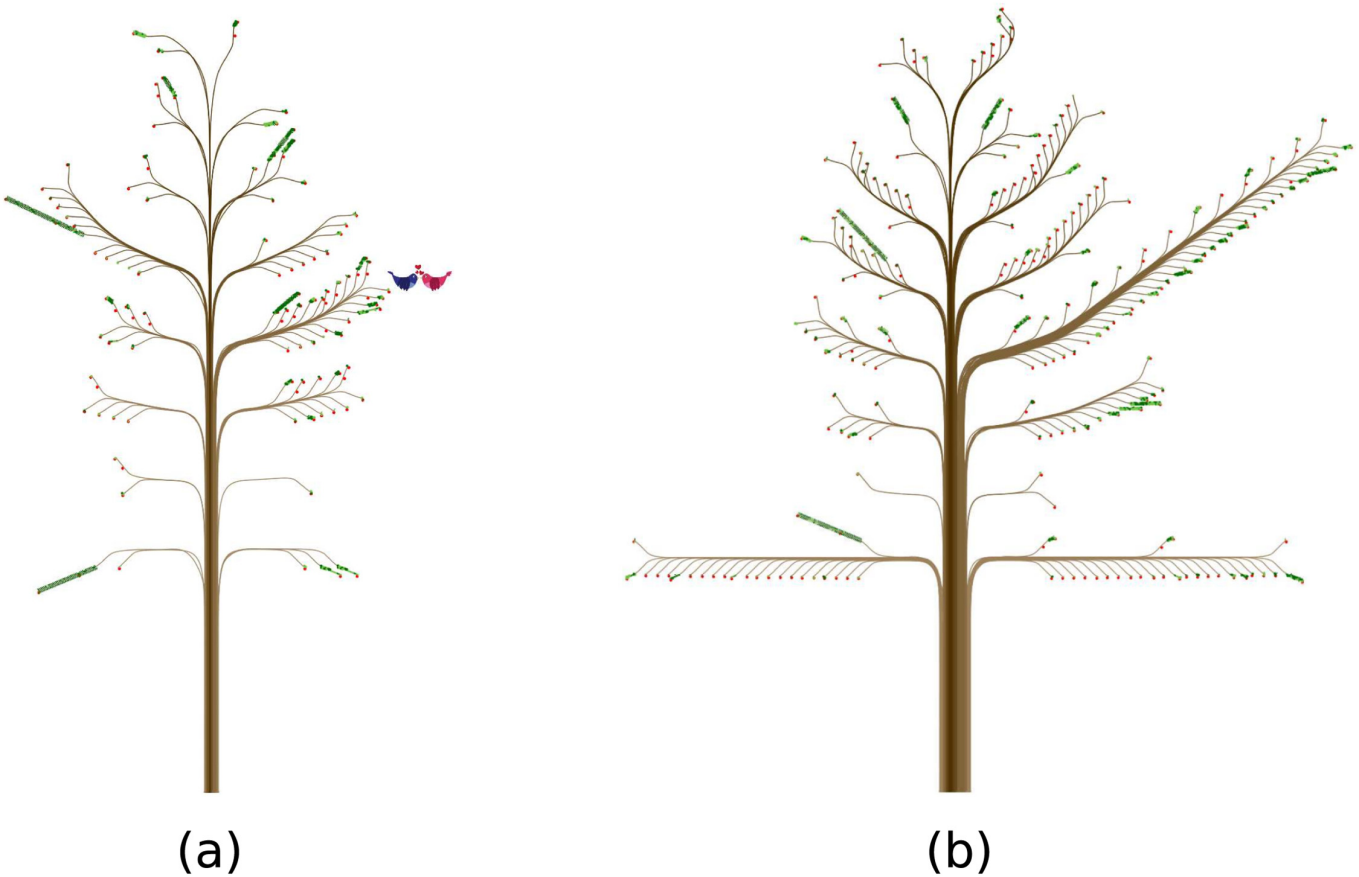
Further stories from *ContactTrees*. (a) shows a woman who has a young child. Four years later, as the child has grown up, the numbers of her ties and contacts have increased (b). This is likely because of her child’s activities: She was meeting many children and probably their parents (especially mothers).

Such fluctuation along one’s life course is not uncommon. In Fig 6, for example, the person represented is a married woman who had a child and did not have a large number of ties and contacts in 2004, a pattern consistent with our last example. We know that the child was between 1 and 4 years old in 2004, because he was represented on the lowest main branch of the left side of the trunk (Fig 6a, S10 File). Four years later (in 2008, Fig 6b, S11 File), the woman’s ties and contacts expanded significantly. This change is likely because of the child’s activities: She was meeting a lot of his friends and probably their parents too (especially mothers). Thus, we can speculate that the decrease of the number of ties and contacts when one has a young child may not persist, as exemplified by the previous case of the married couple.

### Observations

The mapping method of our *ContactTrees* aims to highlight various properties of social relations and interpersonal contacts. Inspired by the botanical metaphor, we incorporate key features of a tree structure, such as length, location, shape, size, and color into our design. Applying these methods to two data sets, we have been able to distinguish how ties and contacts vary by comparing main branches, small branches, leaves, and fruits. Some of the observations we made from these trees can be further verified using other empirical data sources. In the contact diaries dataset, for example, we made several inferences about a person’s overall patterns of ties and contacts with others, and highlighted some trends of social relations throughout his or her life span. When we dig deeper into the original data, and even re-interview those persons whose *contact diaries* were used in our illustrations, some of these observations and hypotheses can be confirmed or modified.

For example, the small branch in Fig 4a that bears the most leaves on a main branch parallel to the one with two birds on the opposite side (left) of the trunk is indeed the focal person’s spouse. The same is true about what we learned from Fig 4b. Likewise, the small branches with the most leaves on the first- and second-bottom main branches in Fig 4 are these focal persons’ children.

Our readings about how trees change over time also turn out to be very close to the actual social life of the woman shown in Fig 6. When her 2-year-old son stayed home all day in 2004, she was actually pretty active by keeping in contact with relatives, neighbors, friends, and strangers. Among her ties, most had no specific relationship with her. Four years later when her son attended the kindergarten, she started to build many new ties and made new contacts with the boy’s schoolmates and their parents (that is, both the indirect ties via her son and the contacts out of such ties increased significantly). Equally important, she still maintained contact with others, even strangers. As a result, the numbers of her ties and contacts increased simultaneously. Compared to the increase in ties, the magnitude of the number of contacts may have contributed more to the visualization effect in the second (larger) tree in Fig 6b.

When we compare social relations over time in other cases, the trends may become too complex to be able to fully understand them just by looking at how trees (by a single design) change in two different years. In Fig 5, for example, the two sets of trees reflect the married couple’s changes in social life over 8 years. In 2004, when the husband (an engineer with an environmental protection firm) was 33 and the wife (a specialist in a telecommunications firm) was 32, the couple was newly wedded and childless. Back then, they each had quite a few contacts with co-workers, friends, weakly tied acquaintances, and even strangers. When they had 2 young children between 2004 and 2008, both of them indeed cut down on some of their former ties because of the new family obligations. The husband had moved to a highly bureaucratic optronics firm, however, where he spent most work hours with machines and a limited number of colleagues; these new job conditions also limited his contacts with others.

While the couple spent more time with their young children in 2008, the wife also reduced her ties with others. She continued to work at the same firm in the same position. Even though the number of her ties decreased sharply from 2004 to 2008, the total number of her contacts dropped only slightly, from 1,307 to 1,239. The significant decrease in ties is clearly visible by comparing the branches in Fig 5d and 5e. In contrast, the slight change in the number of contacts may have been overshadowed by the sharply decreased number of ties. In particular, she retained as many contacts with her colleagues, relatives, and weakly tied acquaintances over the 4 years, even though these contacts were limited to a smaller number of persons. After another 4 years, the number of her contacts increased to 1,674, even though her work conditions had not changed.

Such changes may lead to different implications about our mapping strategies, the botanical metaphor, and substantive issues in social networks. For example, the mapping on the *contact diaries* dataset may be better at capturing the overall structure of gender, age, and relations, while an alternative mapping may be more sensitive to other properties. Further, as our visualizations clearly illustrate, a tree’s general visual effects may lie more in the overall structure of the trunk, main branches, and small branches, than in the number of leaves. Such a tendency of visualization effects may coincide with the overall stress on interpersonal ties in the social network literature. It seems more direct to demonstrate and assess the conditions of a social network by its total size and overall structure. As discussed with regard to the change in Fig 6, however, other properties (especially contacts) may add equally critical information to that structure. Therefore, while branches make up the main structure of the tree, the leaves and fruits give the ties’ details while making the tree more natural, which is fundamental and essential to our botanical metaphor.

## Discussions

The potential effectiveness of our visualization can be evaluated from two perspectives. First, there could be limitations to the algorithm itself and its scalability. Second, our method differs markedly from the previous related network visualizations in its capacity to deal with a more sophisticated egocentric network beyond the usual structure of nodes and edges.

### Limitations of the Algorithm

**Fig 7:**
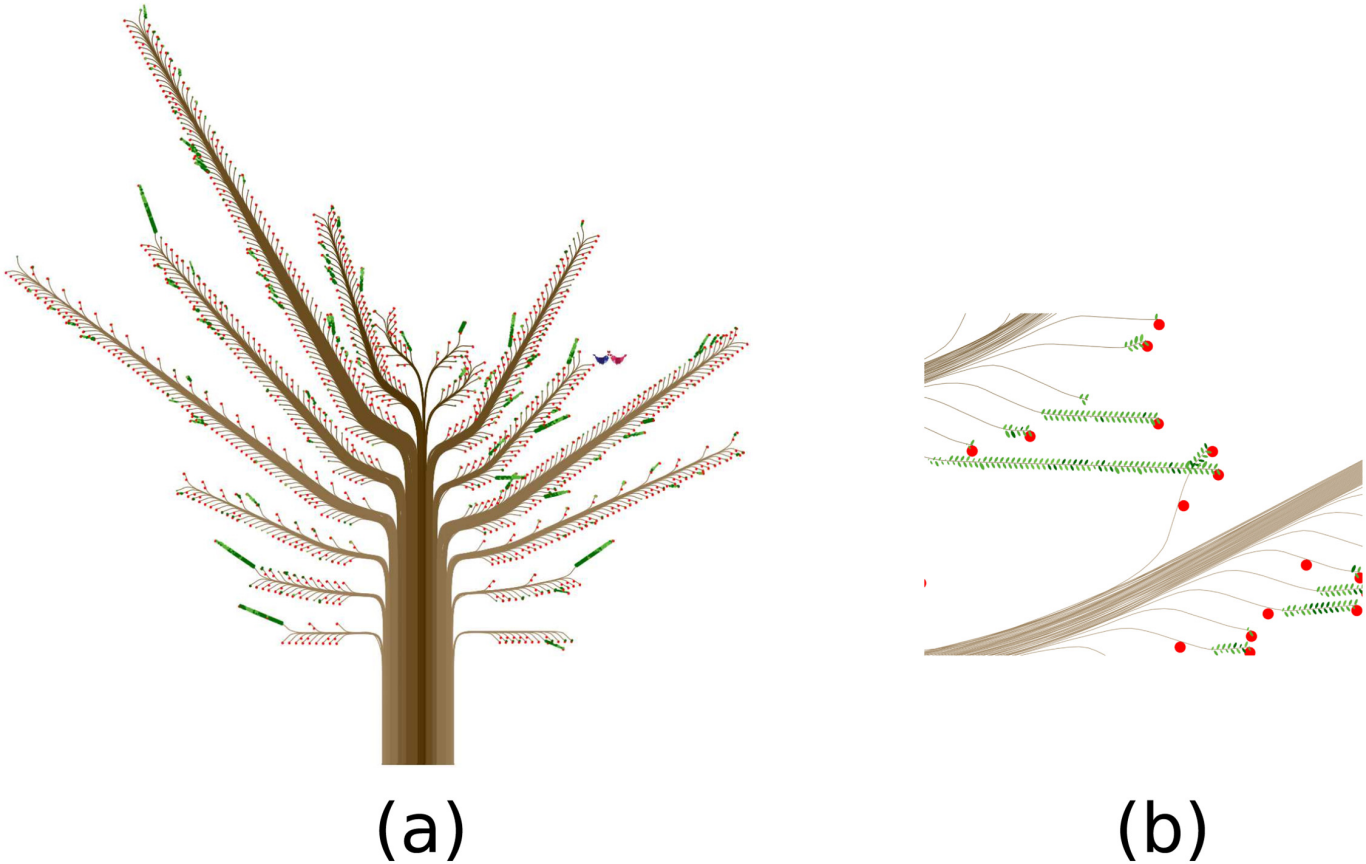
Limitations. (a) This woman has 4,096 contacts (S12 File). Her *ContactTree* is the largest in the *contact diaries* dataset. (b) Two small branches intersect.

Fig 7a shows the woman who has the largest number of contacts in the *contact diaries* dataset. As one can see, the representation is less aesthetically pleasing than those of the previous trees, even though our technique fulfills its purpose: The tree shape is still correct and botanical tree-like, and each feature can be easily distinguished.

Highly unbalanced *ContactTrees* also lead to less aesthetically pleasing representations. Fig 6d shows another example. The woman likes many of her ties very much, and these ties are positioned on the right side of the tree, as indicated by two long main branches. These long branches make the tree less realistic-looking, but it does not disrupt the readability of the data.

The only concrete problem we observed in our datasets concerns branches with many leaves. Sometimes these branches intersect and the leaves overlap, as shown in Fig 7b. Even though this kind of occlusion problem can obscure potentially useful information when one focuses on a specific contact, such intersecting or overlapping is not frequent and does not mislead the user about the global shape of a tree, a main branch, or a small branch. One way to eliminate the problem would be to increase the gap between consecutive main branches, although this also would increase the free space between tree elements.

### ContactTrees vs. Alternative Visualizations

Among the tools designed for visualizing social networks, a “dry” version of dot-line image and *tree-like* network diagrams such as *Vizster* also provide an egocentric view of network structures [13]. Based on the visualization of a node-edge format representing individuals and “friendship” relations, these diagrams offer several interaction techniques that help users select the individuals and ties that they want to visualize or highlight. These diagrams show how the ties of several individuals are related within a large social network by displaying connections among individuals, paths and community structures. In other words, not only do typical egocentric network diagrams show which individuals are linked to the focal person, but they also help identify how the network members are connected with one another. Without a metaphor, however, such visualization tools lack botanic features such as a trunk, main branches, small branches, leaves, and fruits.

The problems that we address are very different from what previous tree-like network diagrams aim to convey. First, we deal with an individual’s ties with his or her network members, but not the ties among these members. As with the case for many other egocentric networks, we do not know whether a network member who is connected to the focal person is also connected to another member. Then, a visualization better suited for complete networks is less compatible in our case.

Second, the *contact diary* dataset includes some information about individuals’ ties that cannot be readily displayed or identified using a typical egocentric network diagram. For example, it would take extraordinary efforts to readjust the tool to show gender and age distributions, as well as who is more favored by the focal person in the same graph. It would be even more challenging to display how these properties evolve over time for a given person. While conventional network visualization tools tend to focus more on showing the whole network, our design can neatly organize and highlight the attributes of each specific tie.

Third, the *contact diary* dataset contains information regarding various attributes about each unique contact between a focal person and the persons he or she encounters. These attributes at the contact level cannot be incorporated into conventional egocentric network diagrams. In comparison, our design takes into account the attributes at both tie and contact levels, thus disclosing the extra and rich information embedded in a sophisticated data structure. In sum, our design enables users to focus on the distribution of contacts across gender, age groups, and different periods of time, while taking into account the sequence of contacts for each individual in terms of contact duration and personal feeling towards each contact, all built into a tree. While other network diagrams typically lack such a time dimension, *ContactTrees* highlights such features as the sequence of interactions that are crucial to the focal person.

A similar botanical visualization tool was proposed [46] to reveal huge hierarchies. The main difference between this tool and our *ContactTrees* lies in the nature of the input data. The purpose of that tool is to visualize a hierarchy, while our tool is intended to visualize a more complex and specific data structure of social networks. Applying that other visualization to this structure would not be a simple project. We would need to find a system for extracting a hierarchy according to ties’ and contacts’ attributes, and we are convinced that designing a specific visualization is more efficient. Moreover, the other visualization has a 3D design and thus induces occlusion issues. The technique used to generate that tree could also help us construct a tree following the same requirements as ours. We decided to use bundled curves instead of cones, however, for several reasons related to the selection of ties.

*TreeVersity* [53] is an interactive visualization that allows users to detect node value changes and topological differences between trees. It was developed to deal with hierarchies, and thus it includes interaction features specifically for this purpose. A *ContactTree* does not represent a hierarchy. There are not internal nodes and the leaves are not unique for each branch. Moreover, our application dataset does not hold information such that a given leaf in a 2004 *ContactTree* corresponds to a particular leaf in a 2008 *ContactTree*, which makes most of the *TreeVersity* comparison techniques less useful for our purpose.

When examining individual variables, conventional statistical graphics such as bar charts and pie charts are often used. These charts are readily available from existing tool kits and do not require much training to design or understand. At times certain “underpowered” visualizations can be redesigned as conventional but more efficient charts (see examples of such redesigns at http://junkcharts.typepad.com/). Nonetheless, while some of these charts may be applied in our context to display the same variables as those highlighted in *ContactTree*, they depict only one variable at a time. For example, to find out “how many ties of a given gender within a certain age range an individual has known for more than 5 years”, one would have to select gender, age, and the number of years known before displaying that right chart. In contrast, with *ContactTrees* the user is able to show all the variables on a single image.

Another advantage of using *ContactTrees* rather than conventional charts is exemplified by a similar recent user study [54] in which authors compare three approaches for visualizing personal Facebook events. The first approach is a timeline chart. The second one is a radial layout based on the metaphor of a firework. The last one is inspired by a bouquet of flowers, which helps design a highly abstract visualization tool with a strong emphasis on aesthetics, as with the case of *ContactTrees*. Even though such abstract visualizations are harder to understand than other tools, they turn out to be the most successful in encouraging users to explore their personal data, and they provide more insight into their own social relationships and interactions. Such abstract visualizations then may be supplemental to conventional charts, because they hold different advantages and disadvantages.

## Conclusion and Future Work

*ContactTrees* make it possible to map ties and contacts based on various priorities and preferences. Facing highly sophisticated network data, whether they are taken from *contact diaries*, citation records, survey archives, or online social media, researchers and users would benefit greatly by first looking at the key features of a tree. These features capture not only “ties,” the underlying unit of analysis in most social-network analyses, but also “contacts” within each tie. Without contacts, we would miss the key information that underlies all social relations. In the botanical terms, without leaves and fruits we could hardly judge a tree’s main characteristics or how it grows over seasonal changes and other life cycles. By better capturing the structure and dynamics of social relations and interpersonal interactions, which in turn facilitate instant comparisons of how network structures differ, the addition of this key property thus distinguishes our design from the majority of other visualization tools for displaying social networks.

To distinguish various branches, including small branches at the tie level, our design takes into account the variables that are critical and essential for understanding egocentric networks. For example, we follow the fundamental building principle behind a population pyramid by using gender and age of contacted persons to construct *ContactTrees*. In addition, we adopt one objective variable, the years of acquaintanceship, and one subjective variable, how much the person likes each of the contacted persons, that help define the strength of ties between the focal person and the network members. At the contact level, we also take both objective and subjective measurements to highlight variations in the appearance of leaves. While leaf sizes vary by the duration of the contact, leaf colors differ by how the person felt about each contact. Other tree-like network visualization tools and systems also make it possible to visualize some of these important features of egocentric networks, but most such systems are composed of nodes and edges only. Other tools may be able to help display some properties of interpersonal contacts, but they tend to show such properties one at a time. By combining these visualizations together, *ContactTrees* is the first system to fill the gap and feature how contacts vary from one another, in addition to showing tie characteristics. Following the botanic metaphor, furnishing leaves and fruits to the trunk and branches greatly enhances the capacities of network visualization tools.

Several features of the design remain to be refined and revised. For one thing, the saturation of lines that make up branches are a potentially attractive feature of the design. When certain small branches are already too thin and light, however, the saturation features make the small branches less visible. Future work may need to address such a trade-off dilemma between aesthetic effects and visibility. Another feature that may not be readily meaningful in the design is the sequences of contacts. Due to the botanic metaphor, the main branches are angled and the sequences of contacts cannot be properly compared. Even though such sequences of contacts are relevant in social network analysis (for example, the properties of subsequent contacts may rely on previous contacts), the factor plays a less important role here, because the emphasis is on conveying the whole picture by visualizing all aspects of ties and contacts at a glance. Furthermore, the positions of both fruits and birds are visual variables that bear no specific meanings. The apparent length of the branches may also be biased due to the building process. To highlight the key characteristics of the ties and the marital status of the focal person and to visualize the overall network structures based on gender and age, we construct these tree features according to current strategies. Future studies could certainly advance such a metaphorical design by polishing these tree features in greater detail.

To further illustrate or clarify how ties and contacts change over time, one may also need to try different mapping methods based on other principles. Because the quality of ties plays a significant role in the overall health of an egocentric network, future work may also want to elaborate how to better display the varying conditions in trunk and branches. Finally, the case studies we use to map our *ContactTrees* were taken from a relatively small number of individuals in a single society. As egocentric networks may differ a great deal by political, economic, and cultural institutions, cross-cultural or cross-national comparisons would further reveal variations in interpersonal ties and contacts [50, 55]. When compatible samples and data become available from other societies or cultures, the design could be applied and further tested for more widespread usefulness.

The use of *ContactTrees* can supplement other formats or sources of network data. Among the abundant resources about social interactions, the booming online social media represent one of the most challenging yet promising sources upon which *ContactTrees* could be applied with ease for both researchers and other end-users. Our design could also be extended to other domains to help identify patterns and trends of social interactions. In particular, alternative formats of *ContactTrees* could show overall ties and contacts for a specific age cohort, occupational group, or the whole population within a geographical area. In that case, our design would facilitate knowledge discovery on social networks at both micro and macro levels, thus expanding potential contributions of network visualization beyond nodes and edges.

## Supporting Information

S1 File Codebook for Data. (TXT)

S2 File Data for Fig 4a. (XLSX)

S3 File Data for Fig 4b. (XLSX)

S4 File Data for Fig 5a. (XLSX)

S5 File Data for Fig 5b. (XLSX)

S6 File Data for Fig 5c. (XLSX)

S7 File Data for Fig 5d. (XLSX)

S8 File Data for Fig 5e. (XLSX)

S9 File Data for Fig 5f. (XLSX)

S10 File Data for Fig 6a. (XLSX)

S11 File Data for Fig 6b. (XLSX)

S12 File Data for Fig 7a. (XLSX)

## References

[1] Wasserman S, Faust K. Social Network Analysis: Methods and Applications. Cambridge University Press; 1994.

[2] Scott JP. Social Network Analysis: A Handbook. SAGE Publications; 2000.

[3] Watts DJ. A Twenty-First Century Science. Nature. 2007;445(489). 17268455 10.1038/445489a

[4] Watts DJ. Six Degrees: The Science of a Connected Age. W. W. Norton and Company; 2004.

[5] Baur M, Benkert M, Brandes U, Cornelsen S, Gaertler M, Köpf B, et al Visone In: Graph Drawing; 2001 p. 463–464.

[6] Borgatti SP, Everett MG, Freeman LC. UCINET: Software for Social Network Analysis;. “Analytic Technologies” (http://analytictech.com/).

[7] Graphviz: Graph Visualization Software;. (http://www.graphviz.org).

[8] Ma KL, Muelder C. Large-Scale Graph Visualization and Analytics. IEEE Computer. 2013;46(7):39–46. 10.1109/MC.2013.242

[9] de Sola Pool I, Kochen M. Contacts and Influence. Social Networks. 1978;1:5–51. 10.1016/0378-8733(78)90011-4

[10] Kadushin C. Understanding Social Networks: Theories, Concepts, and Findings. Oxford University Press; 2012.

[11] Fu YC. Contact Diaries: Building Archives of Actual and Comprehensive Personal Networks. Field Methods. 2007;19(2):194–217. 10.1177/1525822X06298590

[12] Yee KP, Fisher D, Dhamija R, Hearst MA. Animated Exploration of Dynamic Graphs with Radial Layout In: Proceeding of the IEEE Symposium on Information Visualization (InfoVis’01); 2001 p. 43–50.

[13] Heer J, Boyd D. Vizster: visualizing online social networks. In: Proceedings of the IEEE Symposium on Information Visualization (InfoVis’05); 2005. p. 32–39.

[14] Fisher D. Using Egocentric Networks to Understand Communication. IEEE Internet Computing. 2005;9(5):20–28. 10.1109/MIC.2005.114

[15] Fu YC, Ho HC, Chen HM. Weak ties and contact initiation in everyday life: Exploring contextual variations from contact diaries. Social Networks. 2013;35(3):279–287. 10.1016/j.socnet.2013.02.004

[16] Kahneman D, Krueger AB, Schkade DA, Schwarz N, Stone AA. A Survey Method for Characterizing Daily Life Experience: The Day Reconstruction Method. Science. 2004;306(5702):1776–1780. 10.1126/science.1103572 15576620

[17] Freeman LC. Visualizing social networks. Journal of Social Structures. 2000;1(1).

[18] Battista GD, Eades P, Tamassia R, Tollis IG. Graph Drawing: Algorithms for the Visualization of Graphs. Prentive Hall; 1999.

[19] Kaufmann M, Wagner D. Drawing Graph: Methods and Models. Springer, LNCS 2025; 2001.

[20] Herman I, Melançon G, Marshall MS. Graph Visualization and Navigation in Information Visualization: A Survey. IEEE Transactions on Visualization and Computer Graphics. 2000;6(1):24–43. 10.1109/2945.841119

[21] von Landesberger T, Kuijper A, Schreck T, Kohlhammer J, van Wijk JJ, Fekete JD, et al Visual Analysis of Large Graphs: State-of-the-Art and Future Research Challenges. Computer Graphics Forum. 2011;30(6):1719–1749. 10.1111/j.1467-8659.2011.01898.x

[22] Archambault D, Munzner T, Auber D. TopoLayout: Multilevel Graph Layout by Topological Features. IEEE Transactions on Visualization and Computer Graphics. 2007;13(2):305–317. 10.1109/TVCG.2007.46 17218747

[23] Abello J, van Ham F, Krishnan N. ASK-GraphView: A Large Scale Graph Visualization System. IEEE Transactions on Visualization and Computer Graphics. 2006;12(5):669–676. 10.1109/TVCG.2006.120 17080786

[24] van Ham F, van Wijk JJ. Interactive Visualization of Small World Graphs. In: Proceedings of the IEEE Symposium on Information Visualization (InfoVis’04); 2004. p. 199–206.

[25] Muelder C, Ma KL. Rapid graph layout using space filling curves. IEEE Transactions on Visualization and Computer Graphics. 2008;6(14):1301–1308. 10.1109/TVCG.2008.158 18988977

[26] Sallaberry A, Muelder C, Ma KL. Clustering, Visualizing, and Navigating for Large Dynamic Graphs In: Proceedings of the 20th International Symposium on Graph Drawing (GD 2012). LNCS 7704. Springer Berlin Heidelberg; 2013 p. 487–498.

[27] Holten D, van Wijk J. Force-Directed Edge Bundling for Graph Visualization. Computer Graphics Forum. 2009;28(3):983–990. 10.1111/j.1467-8659.2009.01450.x

[28] Henry N, Fekete JD, McGuffin MJ. NodeTrix: a Hybrid Visualization of Social Networks. IEEE Transactions on Visualization and Computer Graphics. 2007;13(6):1302–1309. 10.1109/TVCG.2007.70582 17968078

[29] Lee B, Parr CS, Plaisant C, Bederson BB, Veksler VD, Gray WD, et al TreePlus: Interactive Exploration of Networks with Enhanced Tree Layouts. IEEE Transactions on Visualization and Computer Graphics. 2006;12(6):1414–1426. 10.1109/TVCG.2006.106 17073365

[30] van Ham F, Perer A. Search, Show Context, Expand on Demand: Supporting Large Graph Exploration with Degree-of-Interest. IEEE Transactions on Visualization and Computer Graphics. 2009;15(6):953–960. 10.1109/TVCG.2009.108 19834159

[31] Fisher D, Dourish P. Social and Temporal Structures in Everyday Collaboration. In: Proceedings of the 2004 Conference on Human Factors in Computing Systems (CHI’04); 2004. p. 551–558.

[32] Whittaker S, Jones Q, Nardi BA, Creech M, Terveen LG, Isaacs E, et al ContactMap: Organizing communication in a social desktop. ACM Transactions on Computer-Human Interaction. 2004;11(4):445–471. 10.1145/1035575.1035580

[33] MacLean D, Hangal S, Teh SK, Lam MS, Heer J. Groups Without Tears: Mining Social Topologies from Email. In: Proceedings of the 16th international conference on Intelligent user interfaces (IUI’11); 2011. p. 83–92.

[34] McGuffin MJ, Balakrishnan R. Interactive visualization of genealogical graphs. In: Proceedings of the IEEE Symposium on Information Visualization (InfoVis’05); 2005. p. 16–23.

[35] Bezerianos A, Dragicevic P, Fekete JD, Bae J, Watson B. GeneaQuilts: A System for Exploring Large Genealogies. IEEE Transactions on Visualization and Computer Graphics. 2010;16(6):1073–1081. 10.1109/TVCG.2010.159 20975145

[36] Tuttle C, Nonato LG, Silva C. PedVis: A Structured, Space-Efficient Technique for Pedigree Visualization. IEEE Transactions on Visualization and Computer Graphics. 2010;16(6):1063–1072. 10.1109/TVCG.2010.185 20975144

[37] Jürgensmann S, Schulz HJ. A Visual Survey of Tree Visualization. In: Poster Abstracts of IEEE VisWeek 2010; 2010.

[38] Kruskal JB, Landwehr JM. Icicle plots: Better displays for hierarchical clustering. The American Statistician. 1983;37(2):162–168. 10.2307/2685881

[39] Andrews K, Heidegger H. Information slices: Visualising and exploring large hierarchies using cascading, semi-circular discs. In: Proceedings of the IEEE Symposium on Information Visualization (InfoVis’98); 1998. p. 9–12.

[40] Stasko JT, Zhang E. Focus+context display and navigation techniques for enhancing radial, space-filling hierarchy visualizations. In: Proceedings of the IEEE Symposium on Information Visualization (InfoVis’00); 2000. p. 57–65.

[41] Shneiderman B, Plaisant C. Treemaps for space-constrained visualization of hierarchies; 2009. Http://www.cs.umd.edu/hcil/treemap-history/index.shtml

[42] Auber D, Huet C, Lambert A, Renoust B, Sallaberry A, Saulnier A. GosperMap: Using a Gosper Curve for Laying out Hierarchical Data. IEEE Transactions on Visualization and Computer Graphics. 2013;19(11):1820–1832. 10.1109/TVCG.2013.91 24029903

[43] Ulam S. On some mathematical properties connected with patterns of growth of figures. In: Proceedings of Symposia on Applied Mathematics 14; 1962. p. 215–224.

[44] Honda H. Description of the form of trees by the parameters of the tree-like body: Effects of the branching angle and the branch length on the shape of the tree-like body. Journal of Theoretical Biology. 1971;31:331–338. 10.1016/0022-5193(71)90191-3 5557081

[45] Palubicki W, Horel K, Longay S, Runions A, Lane B, Mech R, et al Self-organizing tree models for image synthesis. ACM Transactions on Graphics. 2009;28(3). 10.1145/1531326.1531364

[46] Kleiberg E, Wetering HVD, Van Wijk J. Botanical Visualization of Huge Hierarchies. In: Proceedings of the IEEE Symposium on Information Visualization (InfoVis’01); 2001. p. 87–94.

[47] Chlan EB, Rheingans P. A Botanically Inspired High-Dimensional Visualization with Multivariate Glyphs. In: Proceedings of the Joint Eurographics/IEEE VGTC Symposium Visualization, VisSym; 2004. p. 231–236.

[48] Stefaner M, Taraborelli D, Ciampaglia GL. Notabilia Visualizing Deletion Discussions on Wikipedia; 2010. Http://notabilia.net/

[49] Xiong R, Donath JS. PeopleGarden: Creating Data Portraits for Users. In: Proceedings of the 12th annual ACM symposium on User interface software and technology; 1999. p. 37–44.

[50] David B, Huszti E, Barna I, Fu YC. Egocentric contact networks in comparison: Taiwan and Hungary. Social Networks. 2016;44:253–265. 10.1016/j.socnet.2015.10.001

[51] Lonkila M. Social networks in post-Soviet Russia: Continuity and change in the everyday life of St. Petersburg teachers. Kikimora Publications; 1999.

[52] Auber D. Tulip—A huge graph visualization framework In: Mutzel P, Jünger M, editors. Graph Drawing Software. Mathematics and Visualization Series. Springer Verlag; 2003.

[53] Gómez JA, Buck-Coleman A, Plaisant C, Shneiderman B. TreeVersity: Interactive Visualizations for Comparing Two Trees with Structure and Node Value Changes. Human-Computer Interaction Lab, University of Maryland; 2012. HCIL-2012-04.

[54] Wang S, Tanahashi Y, Leaf N, Ma K. Design and Effects of Personal Visualizations. IEEE Computer Graphics and Applications. 2015;35(4):82–93. 10.1109/MCG.2015.7426010791

[55] Lin N, Fu YC, Chen CJJ. Social capital in a comparative perspective In: Lin N, Fu YC, Chen CJJ, editors. Social Capital and Its Institutional Contingency: A Study of the United States, China and Taiwan Routledge Press; 2014 p. 3–18.

