# Correction: Contact Trees: Network Visualization beyond Nodes and Edges

**DOI:** 10.1371/journal.pone.0149324

**Published:** 2016-02-09

**Authors:** Arnaud Sallaberry, Yang-chih Fu, Hwai-Chung Ho, Kwan-Liu Ma

The following information is missing from the Funding section: This work is also supported in part by UC Davis RISE program and U.S. National Science Foundation through grants IIS-1528203 and IIS-1320229.
